# Orexins and primary headaches: an overview of the neurobiology and clinical impact

**DOI:** 10.1080/14737175.2024.2328728

**Published:** 2024-03-22

**Authors:** Emily C. Stanyer, Jan Hoffmann, Philip R. Holland

**Affiliations:** a Headache Group, Wolfson Sensory, Pain and Regeneration Centre, Institute of Psychiatry, Psychology & Neuroscience, King’s College London, London, UK; b Sir Jules Thorne Sleep and Circadian Neuroscience Institute, Nuffield Department of Clinical Neurosciences, University of Oxford, Oxford, UK

**Keywords:** Headache, orexin, hypocretin, migraine, sleep

## Abstract

**Introduction:**

Primary headaches, including migraines and cluster headaches, are highly prevalent disorders that significantly impact quality of life. Several factors suggest a key role for the hypothalamus, including neuroimaging studies, attack periodicity, and the presence of altered homeostatic regulation. The orexins are two neuropeptides synthesized almost exclusively in the lateral hypothalamus with widespread projections across the central nervous system. They are involved in an array of functions including homeostatic regulation and nociception, suggesting a potential role in primary headaches.

**Areas covered:**

This review summarizes current knowledge of the neurobiology of orexins, their involvement in sleep-wake regulation, nociception, and functions relevant to the associated symptomology of headache disorders. Preclinical reports of the antinociceptive effects of orexin-A in preclinical models are discussed, as well as clinical evidence for the potential involvement of the orexinergic system in headache.

**Expert opinion:**

Several lines of evidence support the targeted modulation of orexinergic signaling in primary headaches. Critically, orexins A and B, acting differentially via the orexin 1 and 2 receptors, respectively, demonstrate differential effects on trigeminal pain processing, indicating why dual-receptor antagonists failed to show clinical efficacy. The authors propose that orexin 1 receptor agonists or positive allosteric modulators should be the focus of future research.

## 1. Introduction

Primary headache disorders are highly prevalent [1,2] and disabling conditions [3] which significantly impact quality of life [4]. These include migraine, tension-type headache, and several trigeminal autonomic cephalalgias, including cluster headache (CH) [5]. Whilst treatment advances have been made in recent years, these are largely due to the development of anti-CGRP-based therapies [6,7], and the mechanisms of headache initiation and chronification are still relatively unclear [8,9]. There remains a significant need to lessen the burden for those with primary headache disorders, particularly for those individuals who do not respond to current treatments, experience intolerable side effects, or have contraindications [10]. Recently, several novel neuropeptides and neurotransmitter targets have emerged [11] including the orexins [12], which may prove fruitful for the treatment of these disorders.

The hypothalamus is a small diencephalic region involved in homeostatic regulation including body temperature, hormone release, appetite, and arousal [13]. It has reciprocal connections to the thalamus, brainstem periaqueductal gray (PAG), median raphe nuclei, locus coeruleus, and spinal cord, regions which are involved in pain processing, thus, the hypothalamus and associated areas may be responsible for the underlying pathophysiology of headaches and their related features [5]. Indeed, evidence for the involvement of the hypothalamus in trigeminovascular nociception and headache pathophysiology has been provided by several studies [14,15]. For example, the hypothalamus is shown to be active during the premonitory and attack phases of spontaneous and experimentally triggered migraine [16,17] and CH attacks [18]. Alterations in the functional connectivity between the hypothalamus and pain processing regions have also been observed before the beginning of migraine attacks [19–23]. Moreover, the chronobiological features of several headache disorders [24], the potentially sleep-related attacks of migraine [25–28], and the circannual periodicity of CH bouts [29] implicate alterations to the biological clock, which resides in the suprachiasmatic nucleus (SCN) of the hypothalamus, in their pathophysiology. Furthermore, the premonitory symptoms (e.g. thirst, abnormal fatigue, and frequent urination) associated with migraine headaches, and alterations in sleep architecture and quality in migraine [30] are suggestive of perturbed homeostatic regulation [31]. Highlighting the potential importance of altered hypothalamic signaling in setting the threshold for the initiation of primary headaches, including migraine and CH [32].

This review discusses the neurobiology of the hypothalamic neuropeptide system – the orexinergic system and their potential relevance in the underlying mechanisms and treatment of primary headaches.

### 1.1. Orexins

**Figure 1.**
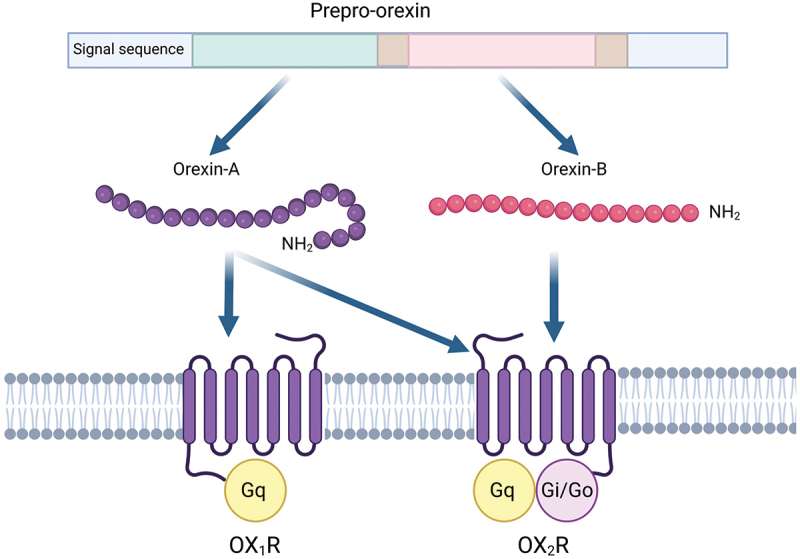
Schematic representation of the orexinergic system.

Although the hypothalamus contains a variety of neurotransmitter and peptide systems including GABA, glutamate, melanin-concentrating hormone, oxytocin, kisspeptin, and neuropeptide Y [33], one important hypothalamic peptide linked to headache pathophysiology is orexin [34]. The orexin system is comprised of two G protein-coupled receptors (GPCR): OX_1_R and OX_2_R (Figure 1). There are two neuropeptides, orexin-A (OXA) and orexin-B (OXB; also known as hypocretin 1 and hypocretin 2) which are cleaved from the precursor protein prepro-orexin [36]. Whilst activation of either receptor is excitatory, the OX_1_R is selective for OXA, whereas OX_2_R is nonselective for OXA and OXB. However, OXB has a 10-fold higher affinity for the OX_2_R than the OX_1_R [37]. The orexin peptides were originally identified for their role in feeding behavior, and consequently termed them the orexins – named after the Greek for appetite [36], while another research group simultaneously named them hypocretin after their hypothalamic location and sequence homology to secretin [38].

The orexin system is comprised of two G-protein coupled receptors (GPCR): orexin-1 (OX_1_R) and orexin-2 (OX_2_R). Two neuropeptides: orexin-A and orexin-B, are cleaved from the precursor protein prepro-orexin. The OX_1_R is selective for OXA, whereas OX_2_R is nonselective for OXA and OXB. However, OXB has a 10-fold higher affinity for the OX_2_R than the OX_1_R. The OX_1_R couples to the G_q_ class of G protein, whereas OX_2_R couples with G_i_ or G_o._ Adapted with permission from PR Holland (2006) [35].

**Figure 2.**
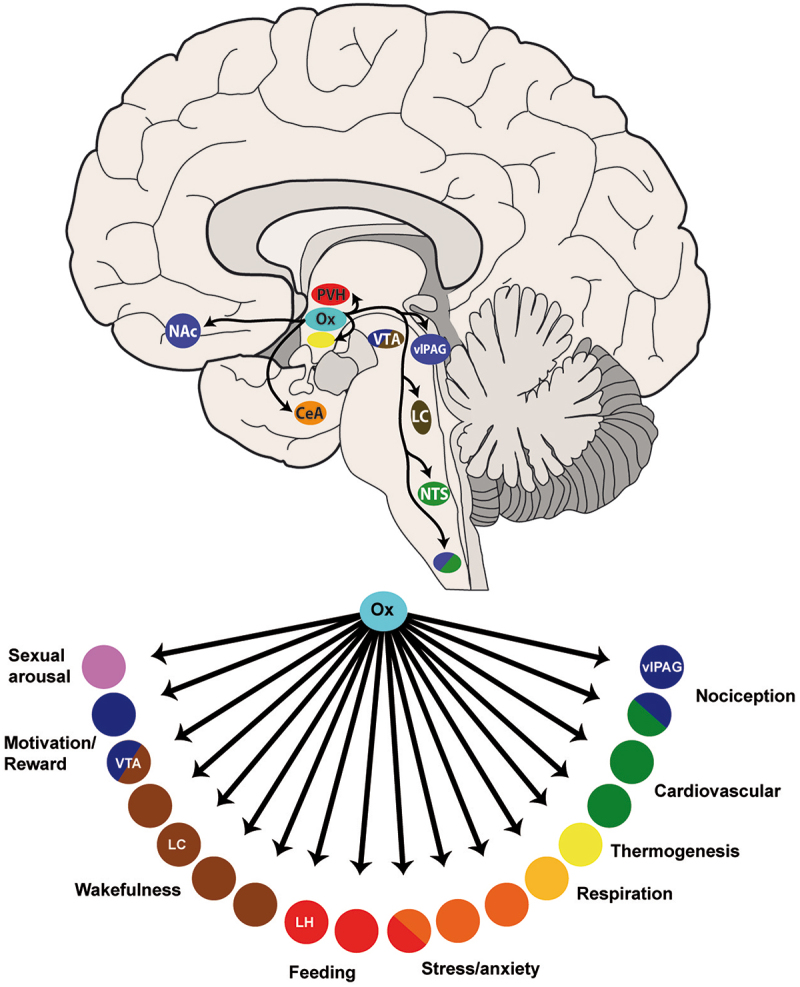
Orexinergic projections and their proposed functions.

The two neuropeptides, OXA and OXB, are largely conserved across rats, mice, and humans [39] and secreted from neuronal cell bodies which are almost exclusively located in the lateral hypothalamus (LH) and perifornical regions of the hypothalamus. The orexinergic neurons represent only ~20% of the neurons in the LH, yet have diffuse projections across the central nervous system (CNS) including to the PAG [40], nucleus accumbens [41], hippocampus [42], thalamus [43], sensory trigeminal neurons [44], and the spinal and trigeminal dorsal horns [42,45], with particularly dense input to the locus coeruleus and tuberomammillary nucleus. In addition to orexin, orexinergic neurons also secrete glutamate [46] and dynorphin [47]. In agreement with the widespread orexinergic projections, orexin receptors are also found to be expressed extensively throughout the CNS [39,48]. This diverse projection pattern and receptor expression has implicated the orexins in a variety of functions from sleep-wake [49], neuroendocrine and autonomic regulation [50], reward-seeking behavior [51], stress [52], feeding behavior [53], and nociception [54] (Figure 2).

The orexins are almost exclusively synthesized in the lateral hypothalamus (LH) and project to many areas of the CNS including brainstem and cortical regions. The orexins are proposed to be involved in a variety of functions from reward, feeding, cognition, to nociception. LC = locus coeruleus; LH = lateral hypothalamus; vlPAG = ventrolateral periaqueductal gray; VTA = ventral tegmental area; Ox = orexin; NTS = nucleus tractus solitarii; NAc = nucleus accumbens; PVH = posterior ventral hypothalamus; CeA = central amygdala. Adapted with permission from P Sureda-Gibert [55].

### 1.2. Orexin in sleep-wake regulation

Although their original discovery was linked to appetite and feeding behavior, the orexins gained further attention for their potential role in sleep-wake regulation and arousal. Initial studies demonstrated that electrical stimulation of the LH led to increased wakefulness, and lesions of the LH resulted in sleep [56,57], leading to their identification as a potential arousal-promoting peptide [58]. Orexinergic neurons inhibit sleep promoting regions including the ventrolateral preoptic (VLPO) nucleus of the hypothalamus [59] to induce wakefulness and conversely GABA-ergic neurons in the VLPO innervate the LH to promote sleep [60]. Direct injection of OXA during the light period (sleep) in rats results in an increase in wakefulness [61] again highlighting their function as a wake-promoting or wake-stabilizing neuropeptide. Orexin is thought to mediate its own expression from neurons by forming a positive-feedback circuit which maintains the orexinergic system at a high level of activity over long periods, resulting in the stabilization of arousal [62], further highlighting their role as wake-stabilizing peptides. In support of this role, several dual-orexin receptor antagonists (DORAs) have been developed and have shown promise in sleep initiation and maintenance, preserving the typical sleep architecture in patients with insomnia [63].

Moreover, support of the orexins’ involvement in sleep/wake regulation came from studies of canine narcolepsy [64]. Narcolepsy is a sleep disorder characterized by inappropriate transitions between sleep and wake [65] as well as daytime somnolence, sleep paralysis, and cataplexy. Narcolepsy patients exhibit an 80–90% reduction in orexinergic neurons in the LH [66] and patients with narcolepsy with cataplexy – narcolepsy type 1 (NT1), demonstrate decreased levels of OXA in cerebrospinal fluid (CSF) [67]. Of interest, narcoleptic patients display an increased prevalence of migraines compared to the general population (females 44.4%, males 28.3% vs 16–25% and 7–8% in the general population) [68], and children with migraines were demonstrated to show a greater risk for developing narcolepsy in a prospective study [69]. A plausible neuroanatomical basis for this may involve diminished orexinergic regulation of PAG, dorsal raphe nucleus (DRN), and locus coeruleus networks. Indeed, these regions are also thought to be involved in the generation, modulation, and cessation of rapid-eye-movement (REM) sleep [70,71], and alterations in REM sleep are reported in narcolepsy [72,73].

More generally, orexin levels in the hypothalamus show circadian variation in mice and humans [74,75] and are maximal during wake, followed by in rapid-eye-movement (REM) sleep and slow-wave sleep [74,76]. Sleep deprivation increases c-Fos activation in orexinergic neurons [77], and sleep disruption is reported to be a key trigger for migraine and CH attacks [78,79].

### 1.3. Orexin’s involvement in nociception/trigeminal nociception

Orexinergic neurons project to the brainstem including the periaqueductal gray (PAG) [80], nucleus raphe magnus (NRM), rostroventromedial medulla (RVM), and superficial lamina of the spinal cord [81]. These regions have been shown to be involved in the trigeminal nociceptive processing [82] suggesting that the orexins may also modulate headache pathophysiology. In particular, the observation that the orexinergic system projects from the hypothalamus to the PAG – a key structure involved in descending pain modulation, also suggests the possibility of modulation of descending inhibitory pathways, or alternatively, through the direct release of orexin at the spinal cord [54,83].

#### 1.3.1. Orexin’s involvement in nociception/pain

The role of the hypothalamus, orexin receptors, and orexin peptides in general nociceptive processing in animal models is contradictory. Direct nociceptive activation excites orexinergic neurons in freely behaving mice [54]; however, both anti-nociceptive and pro-nociceptive effects for orexin have been demonstrated. For example, the stimulation of the hypothalamus can be analgesic [84,85], and inhibits the response of spinal cord neurons to noxious stimulation [86]. Destruction of the ventro-medial-posterior hypothalamus results in transient analgesia [87] and triggers increased neuronal activation in the PAG [88]. In behavioral pain models, both OXA and higher doses of OXB delivered intrathecally resulted in increased hind-paw mechanical withdrawal thresholds in a rat diabetic neuropathic pain model, and this effect was blocked by pre-treatment with an OX_1_R antagonist. However, this was not the case for healthy rats without diabetic neuropathy [89]. In contrast, intrathecal OXA, but not OXB, normalized hind-paw mechanical withdrawal thresholds in rats with sciatic nerve injury [90]. OXA, but not OXB, was analgesic in the hotplate and formalin test [91] in rats and concomitantly decreased the number of c-Fos positive cells in laminae I-II of the spinal cord [81,92] while intracerebroventricular OXA was shown to have antinociceptive effects in assays of thermal, mechanical, and chemical pain [93].

#### 1.3.2. Orexin’s involvement in trigeminal nociception/head pain

Focussing on trigeminal nociception, microinjection of OXA into the posterior hypothalamus inhibits trigeminal nociceptive responses to dural electrical stimulation as well as spontaneous activity, whereas OXB elicited increased responses to stimulation, suggesting a pro-nociceptive role [94]. Similarly, OXA inhibited electrically induced CGRP-dependent vasodilation in an animal model of trigeminovascular activation, and this was reversed by pre-treatment with an OX_1_R antagonist, while OXB had no effect [95]. Whereas conversely, injection of OXA and OXB into the raphe nucleus magnus was shown to facilitate activity in the trigeminal cervical complex, which was thought to be driven mainly by OX_2_R activation [96]. In an animal model of migraine-related mechanical hypersensitivity evoked by the administration of the clinically experimental migraine trigger nitroglycerin (NTG) [97], an OX_1_R antagonist injected into the amygdala resulted in increased anxiogenic responses to NTG in rats, but had no impact on thermal hyperalgesia [98]. In another study, delivery of OXA into the vlPAG attenuated NTG-induced hyperalgesia and photophobia, and this was prevented with administration of an OX_1_R antagonist [99]. Similarly, local OX_1_R antagonism in the vlPAG aggravated NTG-induced anxiety and social conflict, whereas administration of OXA attenuated this effect [100]. Therefore, it is evident that activation of the OX_1_R or administration of OXA may be antinociceptive or involved in preventing associated headache symptomatology, as blocking this receptor seems to attenuate such effects.

The reason for such conflicting results is likely due to the descending inhibitory system being differentially activated during conditions of appropriate stimuli, e.g., under inflammatory or chronic pain conditions and not during acute nociceptive stimuli. Alternatively, the differential functions of orexin receptors could be due to the finding that many brain regions express both OX_1_R and OX_2_R receptors, yet discrete areas including the locus coeruleus show selective expression of a single receptor subtype [101]. Moreover, discrepancies may be due to the location of orexin or antagonist delivery. Its actions in the amygdala may involve the affective components of pain such as stress and anxiety. Indeed, the amygdala may modulate threat learning through the locus coeruleus [102]. This is of interest as headache is co-morbid with anxiety disorders [103], suggesting potential mediation through orexinergic-noradrenergic pathways. In support of this modulation of noradrenergic signaling in the locus, coeruleus has been shown to exert divergent effects on migraine-related cortical and spinal excitation [104]. Thus, the orexinergic system is potentially involved in the modulation of nociceptive transmission and the pathophysiology of headache, but further work is needed to identify the specificity of these pathways and underlying mechanisms.

### 1.4. Orexin and cluster headache

It is widely accepted that the hypothalamus plays a role in CH, with hypothalamic activation observed during attacks [18,105,106]. Initially, reports on a link between CH and the orexin system were circumstantial. This was based predominantly on the striking observation that CH attacks show a clear circadian and circannual rhythmicity, highlighting a potential role of the SCN of the hypothalamus [26]. Specifically, most CH attacks occur at the same time each day, and bouts tend to occur at the same time each year (spring and/or autumn) [27]. However, no significant alterations in the CSF orexin levels in CH have been observed [107], with one study reporting slightly lower levels of orexin [108]. However, CH patients do show endocrinological changes and lack typical circadian regulation of certain hormones which are thought to be under hypothalamic control, such as cortisol, melatonin, prolactin, and testosterone [109].

Studies have also highlighted potential genetic links between orexin and the risk of CH occurrence. The G1246A polymorphism of the OX_2_R gene HCRTR2 is linked to an increased risk of CH [110–112]. Conversely, alternate studies have reported no such link between the G1246A polymorphism and CH [113] which was not linked to treatment response [114], thus the link between genetic orexin factors and CH is unclear. Interestingly, verapamil, a first-line preventive for CH, results in alterations to the hypothalamic CLOCK gene PER2 expression and sleep timing in mice [115]. However, a study demonstrated that the CLOCK gene T3111C polymorphism was not linked to CH [116], suggesting that other hypothalamic CLOCK genes, such as PER2 or those regulating orexin expressions such as DEC2 [117] may be more important in CH pathophysiology.

Another potential link between the orexin system and CH is through REM sleep. Polysomnographic studies of CH have reported reduced REM sleep and longer REM sleep latency compared to healthy controls [118,119]. However, whether this is due to the pain from CH attacks waking up the individuals during the night is unclear, and some studies have shown no link with REM sleep [120]. Orexin is thought to play a modulatory role in REM sleep via the sublaterodorsal tegmental nucleus [121], suggesting a potential link between REM sleep modulation, CH, and orexin.

### 1.5. Orexin and migraine

Given the role of the orexins in homeostatic functions and nociceptive processing, they have also been implicated in migraine pathophysiology. More generally, the hypothalamus is active both prior to and during attacks [16,17], and in the premonitory phase of a migraine attack, the hypothalamus is more responsive to trigeminal nociceptive stimulation [122]. More generally, changes to the light–dark cycle, which may be under hypothalamic control such as jet lag and shift-work are known triggers for attacks [78,123,124], while abnormal fatigue, likely involving dysfunctional arousal regulation, is present in up to 83% of the patients [125]. Moreover, mutations in circadian CLOCK-related genes regulated by the SCN have been associated with increased migraine penetrance in specific families and increased migraine-related phenotypes in preclinical models [126]. Similar to CH, migraine patients also display alterations in sleep architecture, with evidence suggesting reduced REM sleep in both children and adults with migraine [30]. This points to potential hypothalamic orexinergic dysfunction in migraine as the orexins are proposed to play a role in REM sleep modulation.

Evidence for direct alterations of the orexinergic system in migraine, however, is lacking. Higher CSF concentrations of orexin have been reported in chronic migraine [127]. However, this evidence is inconsistent as lower OXA levels have also been observed [128]. Whilst, the HCRTR2 G1246A polymorphism has been potentially linked to CH, studies have shown no significant contribution to the pathophysiology of migraine [129,130]. However, studies have linked the risk of migraine to SNPs in the HCRTR1 gene [131,132].

### 1.6. Orexin’s involvement in accompanying and non-pain symptoms of headaches

Importantly, the hypothalamus has diverse projections and is involved in a range of non-pain related symptoms attributed to headache disorders. For example, patients with migraine may experience associated symptoms such as anxiety, depression, abnormal fatigue, and food cravings, highlighting potential homeostatic dysfunction. The orexins activate the hypothalamic–pituitary axis, which is involved in the body’s response to stress and secretion of hormones [50,133]. Orexins, particularly OXA, are implicated in anxiety behavior [134,135]. Interestingly, CSF OXA levels correlate with anxiety measures in chronic migraine [136], but importantly in this study, the observed orexin levels were not different to controls. This may reflect that only OXA peptide was studied, whereas other studies have looked at orexin levels more generally.

As mentioned previously, the orexins are thought to be involved in feeding behavior and appetite regulation. Descending hypothalamic inputs are integrated with peripheral inputs from the gastrointestinal system [137]. Orexins modulate metabolism as well as nociceptive transmission [138], thus dysfunction to the orexinergic pathway may be a risk factor for co-morbid obesity and migraine [139] as well as being involved in the food cravings associated with migraine attacks.

### 1.7. Clinical impact

Whilst it is evident that orexin may be important in headache pathophysiology and therefore as a potential therapeutic target, very few clinical studies have been conducted based on orexin, despite their approval and efficacy for associated conditions such as insomnia and narcolepsy [63,140,141]. In preclinical models, a precursor of suvorexant, a DORA (DORA-12) was shown to inhibit trigeminal nociception in response to electrical stimulation of dural trigeminal afferents and increased the threshold for inducing cortical spreading depression, the neurophysiological correlate of migraine aura [142], suggesting their potential utility in treating headaches [143]. However, a small clinical trial found no significant reduction in monthly migraine days after the administration of a DORA – filorexant [144]. However, this negative finding could be the result of a variety of factors. For example, the DORA was given at night when orexinergic signaling is the lowest. It is possible that orexin antagonists delivered at other circadian times could be beneficial; however, issues with somnolence persist, supporting the use of selective OX_1_R agonists. Indeed, the orexin itself and peptides involved in headaches such as CGRP show clear diurnal variation [75,145]. Moreover, the short half-life of orexin could be responsible for this null finding. Furthermore, DORAs, as the name suggests, antagonize both orexin receptors, while OXA which preferentially binds to the OX_1_R, has demonstrated antinociceptive actions in preclinical models of migraine, whereas OXB demonstrated largely pronociceptive effects. Therefore, the use of more selective orexin receptor agonists may prove fruitful, especially those targeting the OX_1_R.

Understanding the precise conditions under which orexins are antinociceptive or pronociceptive and the contribution of different orexin receptors to nociception is required to fully elucidate the relationship between orexin, pain, and headaches. Critically, future studies should also explore the most appropriate route/time for delivery, tailored to different individual chronotypes (migraine patients are more likely to have extreme chronotypes that predict attack occurrence timing) [146] and endogenous circadian periods, especially given the symptoms and severity of insomnia (which DORAs were originally developed for) differ depending on chronotype [147]. As such, the antagonism of selective orexin receptors may prove useful in headache patients with co-morbid insomnia, a fact suggested in a post-hoc analysis of clinical data [144]. Chronotherapy studies [148] should be conducted to establish this, thus further research investigating the temporal dynamics of orexin’s antinociceptive properties is warranted.

## 2. Conclusions

Orexins are important neuropeptides involved in sleep-wake regulation and a variety of other homeostatic functions. Their relevance for headache pathophysiology arises from studies of perturbed homeostatic modulation during migraine attacks, genetic links, as well as the circadian and circannual rhythmicity of headache disorders. This rhythmicity points to the role of the SCN which may influence the orexinergic system. The link between the orexin-deficient sleep disorder narcolepsy and headache disorders also suggests a common pathophysiology related to the orexin system. Taken together, the evidence suggests a prominent role of the orexins in headache pathophysiology. However, further research is needed to understand the specificity of individual orexin peptides for nociceptive processing and headache neurobiology. Further large-scale clinical trials of selective orexin receptor agonists, specifically the OX_1_R are required, taking into account circadian fluctuation of endogenous orexin, patient chronotype, and co-morbid sleep disorders.

## 3. Expert opinion

Primary headaches remain one of the most disabling neurological disorders, especially in young women where migraine alone is ranked the number one cause of years lost to disability [149]. The body of evidence available points to a critical role of orexinergic signaling in primary headaches, most prominently migraine and CH, although different receptor subtypes may play a critical role in both. Of particular importance, migraine-related fatigue and the repetitive circannual/circadian rhythmicity of CH remain as key untapped phenotypes in terms of novel therapeutic discovery. Focussing on migraine-related abnormal fatigue, which can occur hours to days before the headache phase [150], it is likely that dysfunctional arousal networks are involved. Patients commonly report feeling washed out and lethargic, which significantly impacts normal everyday tasks and worsens the attack-related disability. Development of novel arousal promoting and antinociceptive compounds, such as OX_1_R agonists or positive allosteric modulators therefore hold significant promise, aided by the recent design and synthesis of such compounds [151], which previously proved unsuccessful. Overall, novel therapies including those targeting calcitonin gene-related peptide have shown some potential in tackling migraine-related fatigue [152], in contrast to the ditans (5-HT_1F_ receptor agonists) which result in somnolence. However, there remains a major therapeutic gap for primary headaches, and we propose that targeted orexinergic modulation is a promising avenue for future clinical development, in conjunction with carefully considered administration routes. Systemic delivery can be hampered by poor CNS penetration, degradation, and unwanted peripheral side effects. This is particularly relevant for intranasal routes which provide rapid CNS access, although this can be impacted by several factors including peptide size and lipophilicity. Indeed, intranasal delivery of orexin A in rats resulted in increased tissue-to-blood ratios when compared to systemic delivery [153], and critically, orexin A levels were found to be highest in the trigeminal nerve, olfactory bulb, and hypothalamus, key areas involved in the pathophysiology of primary headaches.

## Article highlights

- The orexinergic system is postulated to play a role in nociceptive transmission as well as in a range of homeostatic functions including arousal, endocrine regulation, and appetite.
- Modulation of the orexinergic system in preclinical animal models may directly facilitate or attenuate trigeminal nociceptive processing, highlighting the relevance of the orexins in headache pathophysiology.
- Patients with migraine and cluster headache show potential alterations in orexin levels and genetic factors related to the orexinergic pathway.
- There is limited clinical evidence for the efficacy of orexin-based therapeutic targets in primary headaches.
- Future research should focus on selective activation of the orexin 1 receptor via selected routes.

## References

[1] Kopel D, Gottschalk C. The epidemiology of primary headache disorders. Semin Neurol. 2022;42(4):449–458. doi: 10.1055/a-1942-6823 36104164

[2] Onofri A, Pensato U, Rosignoli C, et al. Primary headache epidemiology in children and adolescents: a systematic review and meta-analysis. J Headache Pain. 2023;24(1):8. doi: 10.1186/s10194-023-01541-0 36782182 PMC9926688

[3] Lipton RB, Liberman JN, Kolodner KB, et al. Migraine headache disability and health-related quality-of-life: a population-based case-control study from England. Cephalalgia. 2003;23(6):441–450. doi: 10.1046/j.1468-2982.2003.00546.x 12807523

[4] Abu Bakar N, Tanprawate S, Lambru G, et al. Quality of life in primary headache disorders: a review. Cephalalgia. 2016;36(1):67–91. doi: 10.1177/0333102415580099 25888584

[5] Headache Classification Committee of the International Headache Society (IHS). The international classification of headache disorders, 3rd edition. Cephalalgia. 2018;38(1):1–211. doi: 10.1177/0333102417738202 29368949

[6] Goadsby PJ. Recent advances in understanding migraine mechanisms, molecules and therapeutics. Trends Mol Med. 2007;13(1):39–44. doi: 10.1016/j.molmed.2006.11.005 17141570

[7] Goadsby PJ. Primary headache disorders: five new things. Neurol Clin Pract. 2019;9(3):233–240. doi: 10.1212/CPJ.0000000000000654 31341711 PMC6615655

[8] Buture A, Boland JW, Dikomitis L, et al. Update on the pathophysiology of cluster headache: imaging and neuropeptide studies. J Pain Res. 2019;12:269–281. doi: 10.2147/JPR.S175312 30655693 PMC6324919

[9] Khan J, Asoom LIA, Sunni AA, et al. Genetics, pathophysiology, diagnosis, treatment, management, and prevention of migraine. Biomed Pharmacother. 2021;139:111557. doi: 10.1016/j.biopha.2021.111557 34243621

[10] Brandt RB, Doesborg PGG, Haan J, et al. Pharmacotherapy for cluster headache. CNS Drugs. 2020;34(2):171–184. doi: 10.1007/s40263-019-00696-2 31997136 PMC7018790

[11] Moreno-Ajona D, Villar-Martínez MD, Goadsby PJ Emerging targets for migraine treatment. Neurol India. 2021;69:98. 7 10.4103/0028-3886.315989 34003154

[12] Strother LC, Srikiatkhachorn A, Supronsinchai W. Targeted orexin and hypothalamic neuropeptides for migraine. Neurotherapeutics. 2018;15(2):377–390. doi: 10.1007/s13311-017-0602-3 29442286 PMC5935635

[13] Saper CB, Lowell BB. The hypothalamus. Curr Biol. 2014;24(23):R1111–R1116. doi: 10.1016/j.cub.2014.10.023 25465326

[14] Montagna P. Hypothalamus, sleep and headaches. Neurol Sci. 2006;27(S2):s138–s143. doi: 10.1007/s10072-006-0589-8 16688618

[15] Schulte LH, Allers A, May A. Hypothalamus as a mediator of chronic migraine: evidence from high-resolution fMRI. Neurology. 2017;88(21):2011–2016. doi: 10.1212/WNL.0000000000003963 28446645

[16] Denuelle M, Fabre N, Payoux P, et al. Hypothalamic activation in spontaneous migraine attacks. Headache. 2007;47(10):1418–1426. doi: 10.1111/j.1526-4610.2007.00776.x 18052951

[17] Maniyar FH, Sprenger T, Monteith T, et al. Brain activations in the premonitory phase of nitroglycerin-triggered migraine attacks. Brain. 2014;137(1):232–241. doi: 10.1093/brain/awt320 24277718

[18] Sprenger T, Boecker H, Tolle TR, et al. Specific hypothalamic activation during a spontaneous cluster headache attack. Neurology. 2004;62(3):516–517. doi: 10.1212/WNL.62.3.516 14872051

[19] Moulton EA, Becerra L, Johnson A, et al. Altered hypothalamic functional connectivity with autonomic circuits and the locus coeruleus in Migraine. PloS One. 2014;9(4):e95508. doi: 10.1371/journal.pone.0095508 24743801 PMC3990690

[20] Lerebours F, Boulanouar K, Barège M, et al. Functional connectivity of hypothalamus in chronic migraine with medication overuse. Cephalalgia. 2019;39(7):892–899. doi: 10.1177/0333102419833087 30836766

[21] Meylakh N, Marciszewski KK, Di Pietro F, et al. Altered regional cerebral blood flow and hypothalamic connectivity immediately prior to a migraine headache. Cephalalgia. 2020;40(5):448–460. doi: 10.1177/0333102420911623 32164427

[22] Messina R, Rocca MA, Valsasina P, et al. 2022 Clinical correlates of hypothalamic functional changes in migraine patients [internet]. [cited 2023 May 17]. Available from: https://journals.sagepub.com/doi/full/10.1177/03331024211046618. 10.1177/03331024211046618 34644197

[23] Coppola G, Di Renzo A, Petolicchio B, et al. Increased neural connectivity between the hypothalamus and cortical resting-state functional networks in chronic migraine. J Neurol. 2020;267(1):185–191. doi: 10.1007/s00415-019-09571-y 31606759

[24] Benkli B, Kim SY, Koike N, et al. Circadian features of cluster headache and migraine: a systematic review, meta-analysis, and genetic analysis. Neurology. 2023;100(22):e2224–e2236. doi: 10.1212/WNL.0000000000207240 36990725 PMC10259280

[25] Stanyer EC, Brookes J, Pang JR, et al. Investigating the relationship between sleep and migraine in a global sample: a Bayesian cross-sectional approach. J Headache Pain. 2023;24(1):123. doi: 10.1186/s10194-023-01638-6 37679693 PMC10486047

[26] Engstrøm M, Hagen K, Bjørk M, et al. Sleep-related and non-sleep-related migraine: interictal sleep quality, arousals and pain thresholds. J Headache Pain. 2013;14(1):68. doi: 10.1186/1129-2377-14-68 23919583 PMC3750452

[27] Dexter JD. The relationship between stage III + IV + REM sleep and arousals with Migraine. Headache. 1979;19(7):364–369. doi: 10.1111/j.1526-4610.1979.hed1907364.x 229086

[28] Dexter JD, Riley TL. Studies in Nocturnal migraine. Headache. 1975;15(1):51–62. doi: 10.1111/j.1526-4610.1975.hed1501051.x 1132991

[29] Barloese M, Lund N, Petersen A, et al. Sleep and chronobiology in cluster headache. Cephalalgia. 2015;35(11):969–978. doi: 10.1177/0333102414564892 25573893

[30] Stanyer EC, Creeney H, Nesbitt AD, et al. Subjective sleep quality and sleep architecture in patients with migraine: a meta-analysis. Neurology [Internet]. 2021 [cited 2021 Sep 24;97 16. doi: 10.1212/WNL.0000000000012701 PMC8548957 34551985

[31] Karsan N, Goadsby PJ. Biological insights from the premonitory symptoms of migraine. Nat Rev Neurol. 2018;14(12):699–710. doi: 10.1038/s41582-018-0098-4 30448858

[32] May A, Burstein R. Hypothalamic regulation of headache and migraine. Cephalalgia. 2019;39(13):1710–1719. doi: 10.1177/0333102419867280 31466456 PMC7164212

[33] van den Pol AN, Tsujimoto KL. Neurotransmitters of the hypothalamic suprachiasmatic nucleus: immunocytochemical analysis of 25 neuronal antigens. Neuroscience. 1985;15:1049–1086. doi: 10.1016/0306-4522(85)90254-4 2413388

[34] Holland P, Goadsby PJ. The hypothalamic orexinergic system: pain and primary headaches. Headache. 2007;47(6):951–962. doi: 10.1111/j.1526-4610.2007.00842.x 17578557

[35] Holland PR The orexins and their involvement in the modulation of trigeminovascular nociceptive transmission [Internet] [PhD Thesis]. UCL (University College London); 2006 [cited 2024 Feb 20]. Available from: https://discovery.ucl.ac.uk/id/eprint/1445638/.

[36] Sakurai T, Amemiya A, Ishii M, et al. Orexins and orexin receptors: a family of hypothalamic neuropeptides and G protein-coupled receptors that regulate feeding behavior. Cell. 1998;92(4):573–585. doi: 10.1016/S0092-8674(00)80949-6 9491897

[37] Lang M, Söll RM, Dürrenberger F, et al. Structure−activity studies of orexin a and orexin B at the human orexin 1 and orexin 2 receptors led to orexin 2 receptor selective and orexin 1 receptor preferring ligands. J Med Chem. 2004;47(5):1153–1160. doi: 10.1021/jm030982t 14971895

[38] de Lecea L, Kilduff TS, Peyron C, et al. The hypocretins: hypothalamus-specific peptides with neuroexcitatory activity. Procs Nati Aca Scie. 1998;95:322–327. 10.1073/pnas.95.1.322 PMC18213 9419374

[39] Marcus JN, Aschkenasi CJ, Lee CE, et al. Differential expression of orexin receptors 1 and 2 in the rat brain. J Comp Neurol. 2001;435(1):6–25. doi: 10.1002/cne.1190 11370008

[40] Marcus JN, Elmquist JK. Orexin projections and localization of orexin receptors. In: Nishino S Sakurai T editors. The Orexin/Hypocretin System: physiology and pathophysiology [internet]. Totowa (NJ): Humana Press; 2005 [cited 2023 May 16]p. 21–43. Available from. doi: 10.1385/1-59259-950-8:21

[41] Thorpe AJ, Kotz CM. Orexin a in the nucleus accumbens stimulates feeding and locomotor activity. Brain Res. 2005;1050(1–2):156–162. doi: 10.1016/j.brainres.2005.05.045 15979595

[42] Hervieu GJ, Cluderay JE, Harrison DC, et al. Gene expression and protein distribution of the orexin-1 receptor in the rat brain and spinal cord. Neuroscience. 2001;103(3):777–797. doi: 10.1016/S0306-4522(01)00033-1 11274794

[43] Kirouac GJ, Parsons MP, Li S. Orexin (hypocretin) innervation of the paraventricular nucleus of the thalamus. Brain Res. 2005;1059(2):179–188. doi: 10.1016/j.brainres.2005.08.035 16168969

[44] Stoyanova II, Lazarov NE. Localization of orexin-A-immunoreactive fibers in the mesencephalic trigeminal nucleus of the rat. Brain Res. 2005;1054:82–87. doi: 10.1016/j.brainres.2005.06.066 16054597

[45] Peyron C, Tighe DK, van den AN, et al. Neurons containing hypocretin (orexin) project to multiple neuronal systems. J Neurosci. 1998;18:9996–10015. 23. doi: 10.1523/JNEUROSCI.18-23-09996.1998 9822755 PMC6793310

[46] Rosin DL, Weston MC, Sevigny CP, et al. Hypothalamic orexin (hypocretin) neurons express vesicular glutamate transporters VGLUT1 or VGLUT2. J Comp Neurol. 2003;465(4):593–603. doi: 10.1002/cne.10860 12975818

[47] Chou TC, Lee CE, Lu J, et al. Orexin (hypocretin) neurons contain dynorphin. J Neurosci. 2001;21(19):RC168–RC168. doi: 10.1523/JNEUROSCI.21-19-j0003.2001 11567079 PMC6762880

[48] Trivedi P, Yu H, MacNeil DJ, et al. Distribution of orexin receptor mRNA in the rat brain. FEBS Lett. 1998;438(1–2):71–75. doi: 10.1016/S0014-5793(98)01266-6 9821961

[49] Sakurai T. The neural circuit of orexin (hypocretin): maintaining sleep and wakefulness. Nat Rev Neurosci. 2007;8(3):171–181. doi: 10.1038/nrn2092 17299454

[50] Ferguson AV, Samson WK. The orexin/hypocretin system: a critical regulator of neuroendocrine and autonomic function. Front Neuroendocrinol. 2003;24(3):141–150. doi: 10.1016/S0091-3022(03)00028-1 14596809

[51] Aston-Jones G, Smith RJ, Moorman DE, et al. Role of lateral hypothalamic orexin neurons in reward processing and addiction. Neuropharmacology. 2009;56:112–121. doi: 10.1016/j.neuropharm.2008.06.060 18655797 PMC2635332

[52] Sargin D. The role of the orexin system in stress response. Neuropharmacology. 2019;154:68–78. doi: 10.1016/j.neuropharm.2018.09.034 30266600

[53] Sakurai T. Orexins and orexin receptors: implication in feeding behavior. Regul Pept. 1999;85(1):25–30. doi: 10.1016/S0167-0115(99)00076-2 10588447

[54] Inutsuka A, Yamashita A, Chowdhury S, et al. The integrative role of orexin/hypocretin neurons in nociceptive perception and analgesic regulation. Sci Rep. 2016;6(1):1–15. doi: 10.1038/srep29480 27385517 PMC4935841

[55] Sureda Gibert P Investigating the migraine premonitory phase: neural networks regulating migraine initiation [Internet] [PhD Thesis] KCL (King’s College London); 2022 [cited 2024 Feb 20] Available from: https://kclpure.kcl.ac.uk/ws/portalfiles/portal/249095126/2022_Sureda_Gibert_1309897_ethesis.pdf.

[56] Gerashchenko D, Kohls MD, Greco M, et al. Hypocretin-2-saporin lesions of the lateral hypothalamus produce narcoleptic-like sleep behavior in the rat. J Neurosci. 2001;21(18):7273–7283. doi: 10.1523/JNEUROSCI.21-18-07273.2001 11549737 PMC6762996

[57] Levitt DR, Teitelbaum P Somnolence, akinesia, and sensory activation of motivated behavior in the lateral hypothalamic syndrome. Procs Nati Aca Scie. 1975;72:2819–2823. 10.1073/pnas.72.7.2819 PMC432863 1101268

[58] Alexandre C, Andermann ML, Scammell TE. Control of arousal by the orexin neurons. Curr Opin Neurobiol. 2013;23(5):752–759. doi: 10.1016/j.conb.2013.04.008 23683477 PMC3783629

[59] De Luca R, Nardone S, Grace KP, et al. Orexin neurons inhibit sleep to promote arousal. Nat Commun. 2022;13(1):4163. doi: 10.1038/s41467-022-31591-y 35851580 PMC9293990

[60] Matsuki T, Takasu M, Hirose Y, et al. GABAA receptor-mediated input change on orexin neurons following sleep deprivation in mice. Neuroscience. 2015;284:217–224. doi: 10.1016/j.neuroscience.2014.09.063 25286384

[61] Hagan JJ, Leslie RA, Patel S, et al. Orexin A activates locus coeruleus cell firing and increases arousal in the rat. Procs Nati Aca Scie. 1999;96:10911–10916. 10.1073/pnas.96.19.10911 PMC17982 10485925

[62] Yamanaka A, Tabuchi S, Tsunematsu T, et al. Orexin directly excites orexin neurons through orexin 2 receptor. J Neurosci. 2010;30(38):12642–12652. doi: 10.1523/JNEUROSCI.2120-10.2010 20861370 PMC6633594

[63] Coleman PJ, Gotter AL, Herring WJ, et al. The discovery of suvorexant, the first orexin receptor drug for insomnia. Annu Rev Pharmacol Toxicol. 2017;57(1):509–533. doi: 10.1146/annurev-pharmtox-010716-104837 27860547

[64] Lin L, Faraco J, Li R, et al. The sleep disorder canine narcolepsy is caused by a mutation in the hypocretin (orexin) receptor 2 gene. Cell. 1999;98(3):365–376. doi: 10.1016/S0092-8674(00)81965-0 10458611

[65] Kornum BR, Knudsen S, Ollila HM, et al. Narcolepsy. Nat Rev Dis Primers. 2017;3(1):1–19. doi: 10.1038/nrdp.2016.100 28179647

[66] Thannickal TC, Moore RY, Nienhuis R, et al. Reduced number of hypocretin neurons in human narcolepsy. Neuron. 2000;27(3):469–474. doi: 10.1016/S0896-6273(00)00058-1 11055430 PMC8760623

[67] Peyron C, Faraco J, Rogers W, et al. A mutation in a case of early onset narcolepsy and a generalized absence of hypocretin peptides in human narcoleptic brains. Nat Med. 2000;6(9):991–997. doi: 10.1038/79690 10973318

[68] Dahmen N, Kasten M, Wieczorek S, et al. Increased frequency of migraine in narcoleptic patients: a confirmatory study. Cephalalgia. 2003;23(1):14–19. doi: 10.1046/j.1468-2982.2003.00343.x 12534574

[69] Yang C-P, Hsieh M-L, Chiang J-H, et al. Migraine and risk of narcolepsy in children: a nationwide longitudinal study. PloS One. 2017;12(12):e0189231. doi: 10.1371/journal.pone.0189231 29216286 PMC5720689

[70] Willie JT, Chemelli RM, Sinton CM, et al. Distinct narcolepsy syndromes in orexin receptor-2 and orexin null mice: molecular genetic dissection of non-REM and REM sleep regulatory processes. Neuron. 2003;38(5):715–730. doi: 10.1016/S0896-6273(03)00330-1 12797957

[71] Kantor S, Mochizuki T, Janisiewicz AM, et al. Orexin neurons are necessary for the circadian control of REM sleep. Sleep. 2009;32(9):1127–1134. doi: 10.1093/sleep/32.9.1127 19750917 PMC2737570

[72] Akyildiz UO, Tezer FI, Koc G, et al. The REM-sleep-related characteristics of narcolepsy: a nation-wide multicenter study in Turkey, the REMCON study. Sleep Med. 2022;94:17–25. doi: 10.1016/j.sleep.2022.03.025 35447401

[73] Zhang Y, Ren R, Yang L, et al. Polysomnographic nighttime features of narcolepsy: a systematic review and meta-analysis. Sleep Med Rev. 2021;58:101488. doi: 10.1016/j.smrv.2021.101488 33934047

[74] Taheri S, Sunter D, Dakin C, et al. Diurnal variation in orexin a immunoreactivity and prepro-orexin mRNA in the rat central nervous system. Neurosci lett. 2000;279(2):109–112. doi: 10.1016/S0304-3940(99)00955-6 10674633

[75] Grady SP, Nishino S, Czeisler CA, et al. Diurnal variation in CSF orexin-A in healthy male subjects. Sleep. 2006;29(3):295–297. doi: 10.1093/sleep/29.3.295 16553014

[76] Kiyashchenko LI, Mileykovskiy BY, Maidment N, et al. Release of hypocretin (orexin) during waking and sleep states. J Neurosci. 2002;22(13):5282–5286. doi: 10.1523/JNEUROSCI.22-13-05282.2002 12097478 PMC6758234

[77] Estabrooke IV, McCarthy MT, Ko E, et al. Fos expression in orexin neurons varies with behavioral state. J Neurosci. 2001;21:1656–1662. 5 10.1523/JNEUROSCI.21-05-01656.2001 11222656 PMC6762959

[78] Kelman L. The triggers or precipitants of the acute migraine attack. Cephalalgia. 2007;27(5):394–402. doi: 10.1111/j.1468-2982.2007.01303.x 17403039

[79] Pergolizzi JV, Magnusson P, LeQuang JA, et al. Exploring the connection between sleep and cluster headache: a narrative review. Pain Ther. 2020;9(2):359–371. doi: 10.1007/s40122-020-00172-6 32382871 PMC7648820

[80] Siemian JN, Borja CB, Sarsfield S, et al. Lateral hypothalamic fast-spiking parvalbumin neurons modulate nociception through connections in the periaqueductal gray area. Sci Rep. 2019;9(1):12026. doi: 10.1038/s41598-019-48537-y 31427712 PMC6700312

[81] Bingham S, Davey PT, Babbs AJ, et al. Orexin-A, an hypothalamic peptide with analgesic properties. Pain. 2001;92(1):81–90. doi: 10.1016/S0304-3959(00)00470-X 11323129

[82] Razavi BM, Hosseinzadeh H. A review of the role of orexin system in pain modulation. Biomed Pharmacother. 2017;90:187–193. doi: 10.1016/j.biopha.2017.03.053 28360013

[83] Holland PR, Goadsby PJ. Cluster headache, hypothalamus, and orexin. Current Science Inc. 2009;13(2):147–154. doi: 10.1007/s11916-009-0025-x 19272281

[84] Lopez R, Young SL, Cox VC. Analgesia for formalin-induced pain by lateral hypothalamic stimulation. Brain Res. 1991;563(1–2):1–6. doi: 10.1016/0006-8993(91)91506-V 1786523

[85] Aimone LD, Bauer CA, Gebhart GF. Brain-stem relays mediating stimulation-produced antinociception from the lateral hypothalamus in the rat. J Neurosci. 1988;8(7):2652–2663. doi: 10.1523/JNEUROSCI.08-07-02652.1988 3249250 PMC6569510

[86] Carstens E. Hypothalamic inhibition of rat dorsal horn neuronal responses to noxious skin heating. Pain. 1986;25(1):95–107. doi: 10.1016/0304-3959(86)90012-6 3012442

[87] Millan MJ, Przewlocki R, Millan MH, et al. Evidence for a role of the ventro-medial posterior hypothalamus in nociceptive processes in the rat. Pharmacol Biochem Behav. 1983;18(6):901–907. doi: 10.1016/S0091-3057(83)80013-6 6136987

[88] Behbehani MM, Park MR, Clement ME. Interactions between the lateral hypothalamus and the periaqueductal gray. J Neurosci. 1988;8(8):2780–2787. doi: 10.1523/JNEUROSCI.08-08-02780.1988 2900881 PMC6569401

[89] Kajiyama S, Kawamoto M, Shiraishi S, et al. Spinal orexin-1 receptors mediate anti-hyperalgesic effects of intrathecally-administered orexins in diabetic neuropathic pain model rats. Brain Res. 2005;1044(1):76–86. doi: 10.1016/j.brainres.2005.03.007 15862792

[90] Yamamoto T, Saito O, Shono K, et al. Anti-mechanical allodynic effect of intrathecal and intracerebroventricular injection of orexin-A in the rat neuropathic pain model. Neurosci lett. 2003;347(3):183–186. doi: 10.1016/S0304-3940(03)00716-X 12875916

[91] Heidari-Oranjaghi N, Azhdari-Zarmehri H, Erami E, et al. Antagonism of orexin-1 receptors attenuates swim- and restraint stress-induced antinociceptive behaviors in formalin test. Pharmacol Biochem Behav. 2012;103(2):299–307. doi: 10.1016/j.pbb.2012.08.007 22922083

[92] Yamamoto T, Nozaki-Taguchi N, Chiba T. Analgesic effect of intrathecally administered orexin-A in the rat formalin test and in the rat hot plate test. Br J Pharmacol. 2002;137(2):170–176. doi: 10.1038/sj.bjp.0704851 12208773 PMC1573477

[93] Mobarakeh JI, Takahashi K, Sakurada S, et al. Enhanced antinociception by intracerebroventricularly and intrathecally-administered orexin a and B (hypocretin-1 and -2) in mice. Peptides. 2005;26(5):767–777. doi: 10.1016/j.peptides.2005.01.001 15808907

[94] Bartsch T, Levy MJ, Knight YE, et al. Differential modulation of nociceptive dural input to [hypocretin] orexin a and B receptor activation in the posterior hypothalamic area. Pain. 2004;109(3):367–378. doi: 10.1016/j.pain.2004.02.005 15157698

[95] Holland PR, Akerman S, Goadsby PJ. Orexin 1 Receptor Activation Attenuates Neurogenic Dural Vasodilation in an Animal Model of Trigeminovascular Nociception. J Pharmacol Exp Ther. 2005;315(3):1380–1385. doi: 10.1124/jpet.105.090951 16160082

[96] Supronsinchai W, Hoffmann J, Akerman S, et al. Assessing the quality of health-related quality of life measures. Cephalalgia. 2013;33(4):223–225. SAGE PUBLICATIONS LTD 1 OLIVERS YARD, 55 CITY ROAD, LONDON EC1Y 1SP, ENGLAND. doi: 10.1177/0333102412468681 23223494

[97] Ashina M, Hansen JM, Á Dunga BO, et al. Human models of migraine — short-term pain for long-term gain. Nat Rev Neurol. 2017;13:713–724. 12 10.1038/nrneurol.2017.137 28984313

[98] Askari-Zahabi K, Abbasnejad M, Kooshki R, et al. The role of basolateral amygdala orexin 1 receptors on the modulation of pain and psychosocial deficits in nitroglycerin-induced migraine model in adult male rats. Korean J Pain. 2022;35(1):22–32. doi: 10.3344/kjp.2022.35.1.22 34966009 PMC8728545

[99] Kooshki R, Abbasnejad M, Esmaeili-Mahani S, et al. Activation orexin 1 receptors in the ventrolateral periaqueductal gray matter attenuate nitroglycerin-induced migraine attacks and calcitonin gene related peptide up-regulation in trigeminal nucleus caudalis of rats. Neuropharmacology. 2020;178:107981. doi: 10.1016/j.neuropharm.2020.107981 32745488

[100] Pourrahimi AM, Abbasnejad M, Raoof M, et al. The involvement of orexin 1 and cannabinoid 1 receptors within the ventrolateral periaqueductal gray matter in the modulation of migraine-induced anxiety and social behavior deficits of rats. Peptides. 2021;146:170651. doi: 10.1016/j.peptides.2021.170651 34560171

[101] Boss C, Roch C. Recent trends in orexin research—2010 to 2015. Bioorganic Med Chem Lett. 2015;25(15):2875–2887. doi: 10.1016/j.bmcl.2015.05.012 26045032

[102] Sears RM, Fink AE, Wigestrand MB, et al. Orexin/Hypocretin system modulates amygdala-dependent threat learning through the locus coeruleus. Procs Nati Aca Scie. 2013;110:20260–20265. 10.1073/pnas.1320325110 PMC3864341 24277819

[103] Baskin SM, Lipchik GL, Smitherman TA. Mood and anxiety disorders in chronic headache. Headache. 2006;46(s3):S76–S87. doi: 10.1111/j.1526-4610.2006.00559.x 17034402

[104] Vila-Pueyo M, Strother LC, Kefel M, et al. Divergent influences of the locus coeruleus on migraine pathophysiology. Pain. 2019;160(2):385–394. doi: 10.1097/j.pain.0000000000001421 30371556 PMC6343946

[105] May A, Bahra A, Buchel C, et al. PET and MRA findings in cluster headache and MRA in experimental pain. Neurology. 2000;55(9):1328–1335. doi: 10.1212/WNL.55.9.1328 11087776

[106] May A, Bahra A, Büchel C, et al. Hypothalamic activation in cluster headache attacks. Lancet. 1998;352(9124):275–278. doi: 10.1016/S0140-6736(98)02470-2 9690407

[107] Cevoli S, Pizza F, Grimaldi D, et al. Cerebrospinal fluid hypocretin-1 levels during the active period of cluster headache. Cephalalgia. 2011;31(8):973–976. doi: 10.1177/0333102411403634 21444644

[108] Barloese M. Reduced CSF hypocretin-1 levels are associated. Goo. [cited 2023 May 17]. Available from 10.1177/0333102414562971. 25492975

[109] Strittmatter M, Hamann GF, Grauer M, et al. Altered activity of the sympathetic nervous system and changes in the balance of hypophyseal, pituitary and adrenal hormones in patients with cluster headache. Neuroreport. 1996;7(7):1229–1234. doi: 10.1097/00001756-199605170-00001 8817538

[110] Rainero I, Gallone S, Valfrè W, et al. A polymorphism of the hypocretin receptor 2 gene is associated with cluster headache. Neurology. 2004;63(7):1286–1288. doi: 10.1212/01.WNL.0000142424.65251.DB 15477554

[111] Schurks M, Kurth T, Geissler I, et al. Cluster headache is associated with the G1246A polymorphism in the hypocretin receptor 2 gene. Neurology. 2006;66(12):1917–1919. doi: 10.1212/01.wnl.0000215852.35329.34 16554494

[112] Rainero I, Gallone S, Rubino E, et al. Haplotype Analysis Confirms the Association between the HCRTR2 gene and cluster headache. Headache. 2008;48(7):1108–1114. doi: 10.1111/j.1526-4610.2008.01080.x 18399985

[113] Baumber L, Sjostrand C, Leone M, et al. A genome-wide scan and HCRTR2 candidate gene analysis in a European cluster headache cohort. Neurology. 2006;66(12):1888–1893. doi: 10.1212/01.wnl.0000219765.95038.d7 16801656

[114] Schürks M, Kurth T, Geissler I, et al. The G1246A Polymorphism in the hypocretin receptor 2 gene is not associated with treatment response in cluster headache. Cephalalgia. 2007;27(4):363–367. doi: 10.1111/j.1468-2982.2007.01287.x 17376114

[115] Burish MJ, Han C, Mawatari K, et al. The first-line cluster headache medication verapamil alters the circadian period and elicits sex-specific sleep changes in mice. Chronobiol Int. 2021;38(6):839–850. doi: 10.1080/07420528.2021.1892127 33829951

[116] Cevoli S, Mochi M, Pierangeli G, et al. Investigation of the T3111C CLOCK gene polymorphism in cluster headache. J Neurol. 2008;255(2):299–300. doi: 10.1007/s00415-008-0719-8 18283403

[117] Hirano A, Hsu P-K, Zhang L, et al. DEC2 modulates orexin expression and regulates sleep. Proc Natl Acad Sci, USA. 2018;115(13):3434. doi: 10.1073/pnas.1801693115 29531056 PMC5879715

[118] Barloese MCJ, Jennum PJ, Lund NT, et al. Sleep in cluster headache − beyond a temporal rapid eye movement relationship? Eur J Neurol. 2015;22(4):656–e40. doi: 10.1111/ene.12623 25557272

[119] Marca GD, Vollono C, Rubino M, et al. Dysfunction of arousal systems in sleep-related migraine without aura. 2006;26(7): 857–64. doi: 10.1046/j.1468-2982.2002.00350.x-i1 16776702

[120] Zaremba S, Holle D, Wessendorf TE, et al. Cluster headache shows no association with rapid eye movement sleep. Cephalalgia. 2012;32(4):289–296. doi: 10.1177/0333102411436332 22337861

[121] Feng H, Wen S-Y, Qiao Q-C, et al. Orexin signaling modulates synchronized excitation in the sublaterodorsal tegmental nucleus to stabilize REM sleep. Nat Commun. 2020;11(1):3661. doi: 10.1038/s41467-020-17401-3 32694504 PMC7374574

[122] Schulte LH, May A. The migraine generator revisited: continuous scanning of the migraine cycle over 30 days and three spontaneous attacks. Brain. 2016;139(7):1987–1993. doi: 10.1093/brain/aww097 27190019

[123] Leso V, Gervetti P, Mauro S, et al. Shift work and migraine: a systematic review. J Occup Health. 2020;62(1):e12116. doi: 10.1002/1348-9585.12116 32515906 PMC7154593

[124] Kelman L, Rains JC. Headache and sleep: examination of sleep patterns and complaints in a large clinical sample of migraineurs. Headache. 2005;45(7):904–910. doi: 10.1111/j.1526-4610.2005.05159.x 15985108

[125] Kumar H, Dhamija K, Duggal A, et al. Fatigue, chronic fatigue syndrome and migraine: intersecting the lines through a cross-sectional study in patients with episodic and chronic migraine. J Neurosci Rural Pract. 2023;14:424–431. doi: 10.25259/JNRP_63_2022 37692810 PMC10483198

[126] Brennan KC, Bates EA, Shapiro RE, et al. Casein Kinase Iδ Mutations in familial migraine and advanced sleep phase. Sci, trans med. 2013;5(183):183ra56. doi: 10.1126/scitranslmed.3005784 PMC4220792 23636092

[127] Sarchielli P, Rainero I, Coppola F, et al. Involvement of corticotrophin-releasing factor and orexin-A in chronic migraine and Medication-Overuse Headache: findings from cerebrospinal fluid. Cephalalgia. 2008;28(7):714–722. doi: 10.1111/j.1468-2982.2008.01566.x 18479471

[128] Özaydin göksu E, Özaydin göksu S, Ünal A, et al. Orexin-A levels in episodic and chronic migraine: implications for hypothalamic involvement? J Neurol Sci. 2016;33:56–63.

[129] Pinessi L, Binello E, Martino PD, et al. The 1246 G/A polymorphism of the HCRTR2 gene is not associated with migraine. Cephalalgia. 2007;27(8):945–949. doi: 10.1111/j.1468-2982.2007.01347.x 17645762

[130] Schürks M, Limmroth V, Geissler I, et al. Association between migraine and the G1246A Polymorphism in the hypocretin receptor 2 gene. Headache. 2007;47(8):1195–1199. doi: 10.1111/j.1526-4610.2007.00863.x 17883525

[131] Rainero I, Rubino E, Gallone S, et al. Evidence for an association between migraine and the hypocretin receptor 1 gene. J Headache Pain. 2011;12(2):193–199. doi: 10.1007/s10194-011-0314-8 21344296 PMC3072499

[132] Kowalska M, Kapelusiak-Pielok M, Grzelak T, et al. The new *G29A and G1222A of HCRTR1, 5-HTTLPR of SLC6A4 polymorphisms and hypocretin-1, serotonin concentrations in migraine patients. Front Mole Neurosci [Internet]. 2018;11 [cited 2023 May 18]. Available from: https://www.frontiersin.org/articles/10.3389/fnmol.2018.00191 10.3389/fnmol.2018.00191 PMC5996111 29922128

[133] Watanabe S, Kuwaki T, Yanagisawa M, et al. Persistent pain and stress activate pain-inhibitory orexin pathways. Neuroreport. 2005;16(1):5. doi: 10.1097/00001756-200501190-00002 15618879

[134] Suzuki M, Beuckmann CT, Shikata K, et al. Orexin-A (hypocretin-1) is possibly involved in generation of anxiety-like behavior. Brain Res. 2005;1044(1):116–121. doi: 10.1016/j.brainres.2005.03.002 15862796

[135] Johnson PL, Molosh A, Fitz SD, et al. Chapter 9 - Orexin, stress, and anxiety/panic states. In: Shekhar A, editor. Progress in brain research [internet]: Elsevier; 2012 cited 2023 May 18. p. 133–161. Available from: https://www.sciencedirect.com/science/article/pii/B9780444594891000094 10.1016/B978-0-444-59489-1.00009-4 PMC3665356 22813973

[136] Peres MFP, Vieira DS, Masruha MR, et al. Orexin-A CSF levels correlate with anxiety but not excessive daytime sleepiness in chronic migraine. Headache Medicine. 2011;2:41–45. 2 10.48208/HeadacheMed.2011.9

[137] Zhu J-N, Guo C-L, Li H-Z, et al. Dorsomedial hypothalamic nucleus neurons integrate important peripheral feeding-related signals in rats. J Neurosci Res. 2007;85:3193–3204. 14 10.1002/jnr.21420 17628497

[138] Bigal ME, Lipton RB, Holland PR, et al. Obesity, migraine, and chronic migraine: possible mechanisms of interaction. Neurology. 2007;68(21):1851–1861. doi: 10.1212/01.wnl.0000262045.11646.b1 17515549

[139] Peterlin BL, Rapoport AM, Kurth T. Migraine and obesity: epidemiology, mechanisms, and implications. Headache. 2010;50(4):631–648. doi: 10.1111/j.1526-4610.2009.01554.x 19845784 PMC3969571

[140] Xue T, Wu X, Chen S, et al. The efficacy and safety of dual orexin receptor antagonists in primary insomnia: a systematic review and network meta-analysis. Sleep Med Rev. 2022;61:101573. doi: 10.1016/j.smrv.2021.101573 34902823

[141] Weinhold SL, Seeck-Hirschner M, Nowak A, et al. The effect of intranasal orexin-A (hypocretin-1) on sleep, wakefulness and attention in narcolepsy with cataplexy. Behav Brain Res. 2014;262:8–13. doi: 10.1016/j.bbr.2013.12.045 24406723

[142] Charles A. The migraine aura. Continuum. 2018;24(4):1009. doi: 10.1212/CON.0000000000000627 30074546

[143] Hoffmann J, Supronsinchai W, Akerman S, et al. Evidence for orexinergic mechanisms in migraine. Neurobiol Dis. 2015;74:137–143. doi: 10.1016/j.nbd.2014.10.022 25447225

[144] Chabi A, Zhang Y, Jackson S, et al. Randomized controlled trial of the orexin receptor antagonist filorexant for migraine prophylaxis. Cephalalgia. 2015;35(5):379–388. doi: 10.1177/0333102414544979 25106663

[145] Wimalawansa SJ Circadian variation of plasma calcitonin gene-related peptide in man. J Neuroendocrinology. 1991;3:319–322. 3 10.1111/j.1365-2826.1991.tb00281.x 19215470

[146] van Oosterhout W, van Someren E, Schoonman G, et al. Chronotypes and circadian timing in migraine. Cephalalgia. 2018;38:617–625. doi: 10.1177/0333102417698953 28944680 PMC5896690

[147] Ong JC, Huang JS, Kuo TF, et al. Characteristics of Insomniacs with self-reported morning and evening chronotypes. J Clin Sleep Med. 2007;3(3):289–294. doi: 10.5664/jcsm.26801 17561599 PMC2564777

[148] Cardinali DP, Brown GM, Pandi-Perumal SR, et al. Chapter 24 - chronotherapy. In: Swaab D, Kreier F Lucassen P, editors. Handbook of clinical neurology [internet]: Elsevier; 2021 cited 2023 Feb 17. p. 357–370. Available from: https://www.sciencedirect.com/science/article/pii/B9780128199756000236 10.1016/B978-0-12-819975-6.00023-6 34225975

[149] Steiner TJ, Stovner LJ, Jensen R, et al. Migraine remains second among the world’s causes of disability, and first among young women: findings from GBD2019. J Headache Pain. 2020;21(1):137. doi: 10.1186/s10194-020-01208-0 33267788 PMC7708887

[150] Giffin NJ, Ruggiero L, Lipton RB, et al. Premonitory symptoms in migraine: an electronic diary study. Neurology. 2003;60(6):935–940. doi: 10.1212/01.WNL.0000052998.58526.A9 12654956

[151] Iio K, Hashimoto K, Nagumo Y, et al. Design and synthesis of orexin 1 receptor-selective agonists. J Med Chem. 2023;66(8):5453–5464. doi: 10.1021/acs.jmedchem.2c01773 37043436

[152] Ament M, Day K, Stauffer VL, et al. Effect of galcanezumab on severity and symptoms of migraine in phase 3 trials in patients with episodic or chronic migraine. J Headache Pain. 2021;22(1):6. doi: 10.1186/s10194-021-01215-9 33549036 PMC7868011

[153] Dhuria SV, Hanson LR, Frey WH Intranasal drug targeting of hypocretin-1 (orexin-A) to the central nervous system. J Pharm Sci. 2009;98:2501–2515. 7 10.1002/jps.21604 19025760

